# A healthcare workers’ mHealth adoption instrument for the developing world

**DOI:** 10.1186/s12913-022-08592-0

**Published:** 2022-10-02

**Authors:** Michael Addotey-Delove, Richard E. Scott, Maurice Mars

**Affiliations:** 1 Department of TeleHealth, College of Health Sciences, University of KwaZulu–Natal, 5th Floor Desmond Clarence Building, 238 Mazisi Kunene Rd., Glenwood, KwaZulu-Natal Durban, South Africa; 2 Department of Community Health Sciences, Cumming School of Medicine, University of Calgary, Calgary, Alberta Canada; 3 College of Nursing and Health Sciences, Flinders University, Adelaide, SA Australia

**Keywords:** mHealth, Adoption, Healthcare worker, Assessment scale, eHealth, Telemedicine, Developing world

## Abstract

**Introduction:**

Healthcare workers’ adoption of mHealth is critical to the success or failure of clinician based mHealth services in the developing world. mHealth adoption is affected or promoted by certain factors, some of which are peculiar to the developing world. Identifying these factors and evaluating them will help develop a valid and reliable measuring instrument for more successful prediction of mHealth adoption in the future. The aim of this study was to design and develop such an instrument.

**Method:**

A Healthcare workers’ mHealth Adoption Questionnaire (HmAQ) was developed based on five constructs identified through a prior literature review: multi-sectorial engagement and ownership; staffing and technical support; reliable infrastructure; usefulness and stewardship; and intention to adopt. After testing face and content validity, the questionnaire was administered to 104 nurses and midwives in the Ewutu-Senya district of the Central Region of Ghana who used a maternal mHealth intervention. After data collection confirmatory factor analysis and structural equation modelling were applied and the Healthcare Worker mHealth Adoption Impact Model (HmAIM) developed.

**Results:**

Exploratory factor analysis showed the eigenvalue of all five components to be significant (cumulative total greater than 1.0). Bartlett’s Test of Sphericity was significant, the Kaiser-Meyer-Olkin value was 0.777, and the mean Cronbach’s α value was 0.82 (range 0.81–0.83). Confirmatory factor analysis showed that constructs for the HmAQ were within acceptable limits and valid. Structural equation modelling showed the causal relationships between components. This resulted in development of the HmAIM. A modified model was then developed using the averages of individual construct items. This model showed strong correlation among the constructs. Further research will be required to understand new dimensions of mHealth adoption as a result of emerging technology needs, new complexities in the healthcare work environment, and how different cadres of healthcare workers respond to it.

**Conclusion:**

The study presents a valid and reliable instrument, the HmAIM, to serve as a tool for assessment of healthcare workers’ mHealth adoption in the developing world. Use of the instrument will enhance the likelihood of successful adoption of mHealth implementations.

**Supplementary Information:**

The online version contains supplementary material available at 10.1186/s12913-022-08592-0.

## Introduction

The World Health Organization defines mHealth as the use of mobile devices and wireless technology in healthcare to support objectives of healthcare [1]. mHealth can be used to support healthcare workers, register and manage patients records, make a timely informed decision about care, and communicate with other practitioners [2, 3]. mHealth promotes networking and information sharing, creates a new interactive pathway for patients and healthcare providers, and enhances the way healthcare is delivered [2, 4–6]. However, the optimal benefits of mHealth implementations will not be realised unless people broadly adopt and use them.

In the context of this study, mHealth focusses on the use of mobile devices and an app for the delivery of care to patients by healthcare workers (MoTECH). Of note is that healthcare workers anticipate certain negative aspects of their use of mHealth for clinical interaction with patients [2, 7–10].

Thus, digital devices like mobile phones can both positively and negatively impact healthcare workers [7], and there is the need to identify factors promoting or impeding adoption in order to facilitate acceptance of mHealth among healthcare workers. Adoption remains a major concern for both researchers and practitioners and is considered pivotal to the success or failure of new implementations [11, 12].

The issues determining user acceptance or adoption are usually assessed using models, theories, or frameworks. Existing models and theories that have been used to explain user acceptance of digital implementations in healthcare include the Technology Acceptance Model (TAM) [13], the Unified Theory of Acceptance and Use of Technology (UTAUT) [14], the Theory of Reasoned Action (TRA) [15], the Theory of Planned Behaviour (TPB) [16], the Diffusion of Innovation Theory (DOI) [17], and the Model of Personal Computer Utilization (PCU) [18]. Each of these models use different factors to predict possible use of technology or information systems. The two most commonly used models for performing such assessments are TAM and UTAUT, both of which aim to explain why a technology may be accepted or rejected by users [11].

The original TAM focuses on technology usefulness and its ease of use [19]. Perceived usefulness addresses the user’s expectation that the system will be useful for a particular job. While perceived ease of use addresses the user’s expectation of the system being user-friendly and easy to use. Recent reviews of the use of TAM theories for technology acceptance research in healthcare shows that it accounts for 30–40% of variance of technology acceptance [20]. A number of TAM extensions have been proposed to improve predictive power, such as TAM 2 and TAM 3 [21].

TAM 2 added ‘social influence’ (from the concept of the subjective norm) and ‘the cognitive instrumental process’ (job relevance, output quality, result demonstrability and perceived ease of use) to TAM [22–24]. TAM 3 introduced additional elements such as computer self-efficacy, computer anxiety or enjoyment, and perceptions of external control [25–27].

UTAUT is based on the analysis and consolidation of eight models [14]. The aim was to improve on the capabilities of the original TAM by adding two additional factors explaining the impact of technical and organisational infrastructure and social influence on computer usage behaviour [28–30].

TAM and UTAUT models have explained between 12 and 95% (average ~ 54%) of the variance in behavioural intention to use technology or an information system. However, there are some relationships explained in the models that are not applicable in all contexts. There are also relationships and constructs that are omitted that could better help explain technology acceptance and use in some disciplines [11]. Both TAM and UTAUT have received criticism for limited explanatory and predictive power [31], complexity as well as grouping and labelling of constructs [32, 33], and disregard of settings that require or force users to adopt technology [34].

The latter is of particular importance. Existing predictive frameworks assume that the end-user, in this case healthcare workers, have a choice as to whether they will adopt a new mHealth technology. Often this is not the case. In the developing world mHealth solutions are frequently ‘imposed’ on healthcare workers by governments, institutions, funders/donors, and even other health professionals. There may be no choice, with use of the solution being considered a job requirement. Conversely, during the COVID-19 pandemic many health professionals have spontaneously adopted mHealth solutions without any formal planning or implementation. These mHealth solutions have included instant messaging, mobile phone consultations, and also smartphone-based video-conferencing (using freely available software such as Skype and ZOOM) [35].

It is posited that the constructs in TAM and UTAUT models may not be applicable in all contexts, and that there is the need to develop an alternative model whose constructs are empirically based and context-specific to healthcare workers in the developing world. A recent literature review identified (from empirical studies of mHealth implementations in the developing world) seven constructs for optimum adoption of mHealth by healthcare workers [36]. The overall findings from prior research [36] suggest that multi-sectoral engagement and phone ownership, staffing and technical support, reliable infrastructure, and usefulness and stewardship are key determinants of intention to adopt mHealth by healthcare workers in the developing world.

Based on this prior literature review a novel approach was considered for development of a predictive framework for mHealth adoption. Rather than assessing people’s perceptions of their potential use of a technology, testing of the reliability and validity of the framework would apply views of people who had been required to use the technology. Thus, it was hypothesised that when healthcare workers receive support that addresses the factors identified [36] adoption of mHealth by healthcare workers in the developing world will be facilitated. It was additionally hypothesised that there exist causal relationships between the variables. Thus, the aim of the study was to design and develop a valid and reliable instrument for modelling factors identified to impact mHealth adoption by healthcare workers in the developing world.

## Methods

The methods used to develop the new tool, the Healthcare workers’ mHealth Adoption Questionnaire (HmAQ), and to test its reliability and validity are first described. As the instrument was found to be valid and reliable the methods used for the development of the model, the Healthcare workers mHealth Adoption Impact Model (HmAIM), are then described. Detailed results of the instrument development process are presented in the Results.

### HmAQ development

The questionnaire is intended to identify factors that impact healthcare workers’ adoption of mHealth in the developing world. A 20-item healthcare workers’ mHealth adoption questionnaire (HmAQ) was developed based on results from a recent literature review of factors affecting healthcare worker adoption of mHealth in the developing world [36]. The seven factors identified in the review were consolidated into five constructs for the questionnaire: multi-sectoral engagement and ownership, staffing and technical support, reliable infrastructure, system’s utility, and intention to adopt.

The items in the questionnaire were drafted based on the structure and format of items from three published usability questionnaires, the Telehealth Usability Questionnaire [37], Telemedicine Satisfaction Questionnaire [38], and Patients’ Telecare Assessment Questionnaire [39]. The constructs and their related items used in the questionnaire are listed in Table 1. A 7-point Likert scale was used, with responses ranging from strongly disagree to strongly agree. Face validity of HemAQ was assessed by ten health workers and their responses were used to revise the draft, which was then evaluated by three experts in the field for content validity.

**Table 1:** Listing of the constructs, their focus and related items of the HmAQ

Construct	Addresses the attitude of health workers towards mHealth based on:	Items
1. Multi-sectoral engagement and ownership	The availability of government support, funding, and the supply of mHealth devices to health workers	MFO1-MFO4
2. Staffing and technical support	The availability of appropriate training and technical support, motivation and adequate staffing	HTS1-HTS4
3. Reliable infra-structure	The availability of reliable infrastructure	RI1-RI4
4. System’s utility	The system effectiveness, ease of use, and patients’ access to mobile devices	USC1-USC4
5. Intention to adopt	The availability of multi-sectoral engagement and ownership, staffing and technical support, reliable infrastructure, and the system’s utility	IA1-IA4

### Sample

Convenience sampling was used to select 104 participants, comprising 101 nurses and 3 midwives from 17 Health Posts in the Ewutu-Senya district of the Central Region of Ghana in October 2017 where MoTeCH (Mobile Technology for Community Health (MoTeCH) was being used. All the nurses were female with a minimum of a tertiary level education and who had used MoTeCH for at least 6-months. The nurses were contacted during workshops and seminars organised by the district health directorate, and during child welfare clinics at the various health posts and in the communities. The questionnaire was given to nurses who agreed to participate and was self-administered.

Sample size was estimated based upon guidance from Guadagnoli and Velicer [40] who recommended a minimum adequate sample size of 5 participants per question. This was the minimum number of variables for a component that must be selected to load highly on their main factors in factor analysis, and resulted in a minimal sample size of 100. The appropriateness of the minimum number of variables was determined by observing the Kaiser-Meyer-Olkin (KMO) value which is a measure of the appropriateness of variance among variables in the study that might be common [41]. The sample size was determined to be 110, and a drop-out rate of 10% was assumed.

### Data analysis

Cronbach’s alpha was used to test the internal reliability of the questionnaire. Bartlett test of sphericity was used to check for the existence of redundancy between variables that can be summarised together. It assumed a null hypothesis that the correlation matrix has an identity matrix. Bartlett’s test was significant with a value less than 0.05 indicating that a factor analysis may be useful with the data [42]. Confirmatory factor analysis (CFA) was conducted to show causal relations between latent factors and their observed indicator variables. The results from the CFA showed that a structured equation model could be developed.

### Development of the structural equation model

The data were analysed and convergent, discriminant and nomological validity were all established (Additional file 1: Appendix 2). A structural equation model was then developed to observe the relationships existing between constructs in the instrument developed after all the assumptions of multiple linear regression analysis were satisfied. AMOS 23 software was used to run the analysis and the results shown in (Additional file 1: Appendix 3). Subsequently a modified model was then developed using the averages of individual construct items.

### Ethical approval

Ethical approval for the study was granted by the Biomedical Research Ethics Committee of the University of KwaZulu-Natal and the Ghana Health Service Ethics Review Committee, and participants gave written informed consent.

## Results

### HmAQ development

The HmAQ statistics had a mean estimate of 116.5, variance of 44.02, and standard deviation of 6.7 for the 20-item questionnaire. The differences between the estimated mean for all questions was 116.5 (range 124.8 to 111.1), indicating the items appropriately assessed what was intended. The Pearson correlation showed positive correlation among the constructs with the strongest occurring at 0.490 between USC and IA. All the constructs in the HmAQ showed an appreciable level of correlation between them with a relatively stronger one occurring between HTS and IA.

The Cronbach’s alpha for HmAQ was 0.82, indicating good internal consistency among the items [43]. The KMO measure of sampling accuracy was 0.77, which is higher than the 0.6 suggested by Kaiser as being acceptable [44], and Bartlett’s test of sphericity was significant, *p* < 0.005, for a Chi-Square value of 2253.95 indicating that the constructs were valid and suitable for principal component analysis.

**Fig. 1:**
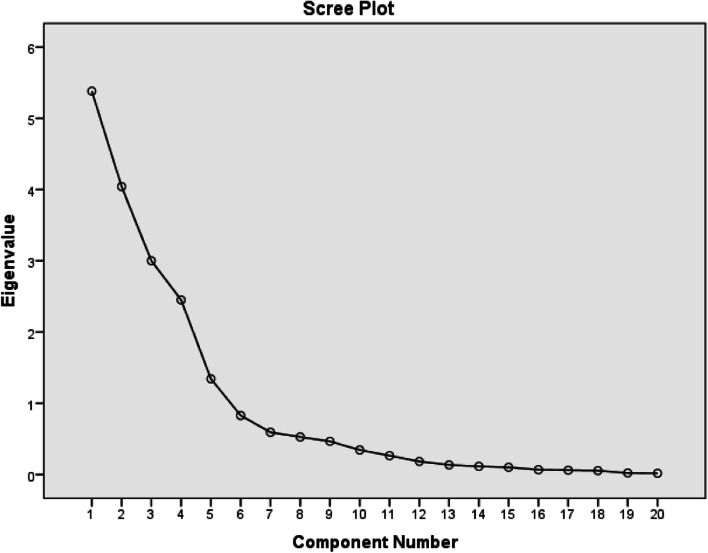
Scree plot showing distribution of factors by their eigenvalues for healthcare workers’ components

An elbow in the Scree plot (Fig. 1) occurs at eigenvalues below one, consequently a threshold of one was chosen for the factor retention cut-off, maximising the variance accounted for. The scree plot shows that of the 20 factorial components, five unrelated components had eigenvalues greater than one (5.382, 4.042, 3.002, 2.451, and 1.344), and accounted for 81.8% of the accumulated variance (Fig. 1). As a consequence these five components were retained for principal component analysis.

### Findings from the HmAQ

Based on the results from the principal component analysis the HmAQ was considered valid and reliable. The results in Additional file 1: Appendix 1 show the component analysis of the factor loadings of the various items loading under specified factors, with the minimum extraction occurring with item RI2 = 0.463 and the highest extraction occurring at RI1 = 0.970. The average extraction of all the HmAQ items was 0.850 which is high and indicates that the factor loadings were appropriate for all items.

The factor loadings produced after Principal Component Analysis were based on the healthcare workers’ response as presented by the factor analysis. The HTS questions had a high loading of 0.922 and above, and loaded mainly on the first axis-factor, with eigenvalue 5.382, which explains nearly 27% of the total variance. The first factor loading represents the attitude of healthcare workers towards mHealth use based on the availability of appropriate training and technical support, motivation and adequate staffing. This factor highlights the availability of appropriate training and technical support, motivation, and adequate staffing as the main components influencing the adoption of mHealth by healthcare workers. The reliability of the first factor was 0.950, which was satisfactory.

The second construct had the USC questions and showed a high factor loading with loading above 0.861on the second component axis, and an eigenvalue of 4.042, explaining a little over 20% of the total variance accumulated. The second construct’s questions represent the attitude of healthcare workers towards mHealth adoption based on the availability of system effectiveness, ease of use, and patient’s access to mobile devices. The reliability for this factor was 0.920 which is satisfactory.

The third construct questions items, IA1, IA2, IA3, and AI4, loaded high with factor loadings and load on the third axis respectively with an accompanying eigenvalue of 3.002 and explain 15% of the total variance accumulated. This question represents the intention of healthcare workers towards adopting mHealth in future. The reliability of this factor was 0.888 which is satisfactory.

The fourth construct question items (RI1, RI3, and RI4) loaded with high factor loadings above 0.950 on the fourth component axis with an accompanying eigenvalue of 2.451, which explains a little over 12% of the total variance accumulated. This construct’s questions represent the attitude of healthcare workers towards mHealth use based on the availability of reliable infrastructure. The reliability of this factor of 0.880 is satisfactory.

The fifth and final construct has item questions MFO1, MFO2, MFO3, and MFO4 showing a moderately high factor loading on the fifth component axis, with an eigenvalue of 1.344 and explaining about 7% of the total variance accumulated. This constructs’ questions represent the attitude of healthcare workers towards mHealth use based on the availability of appropriate training, motivation and related matters. This highlights collaboration and funding as the least important component influencing healthcare workers’ adoption of mHealth. The reliability was 0.842 which is satisfactory.

Given the above results the HmAQ, with five constructs, has been developed and validated. Further work can be carried out on this questionnaire to investigate how different health worker groupings respond to it. The generalised weighted least square method was used to determine if the model fits well. The significance sign for the goodness of fit test from the Principal Component Analysis of the Exploratory Factor Analysis from the model development was 0.084: this is greater than 0.05 – the cut-off point – allowing the null hypothesis to be accepted.

### Development of the structural equation model

**Fig. 2:**
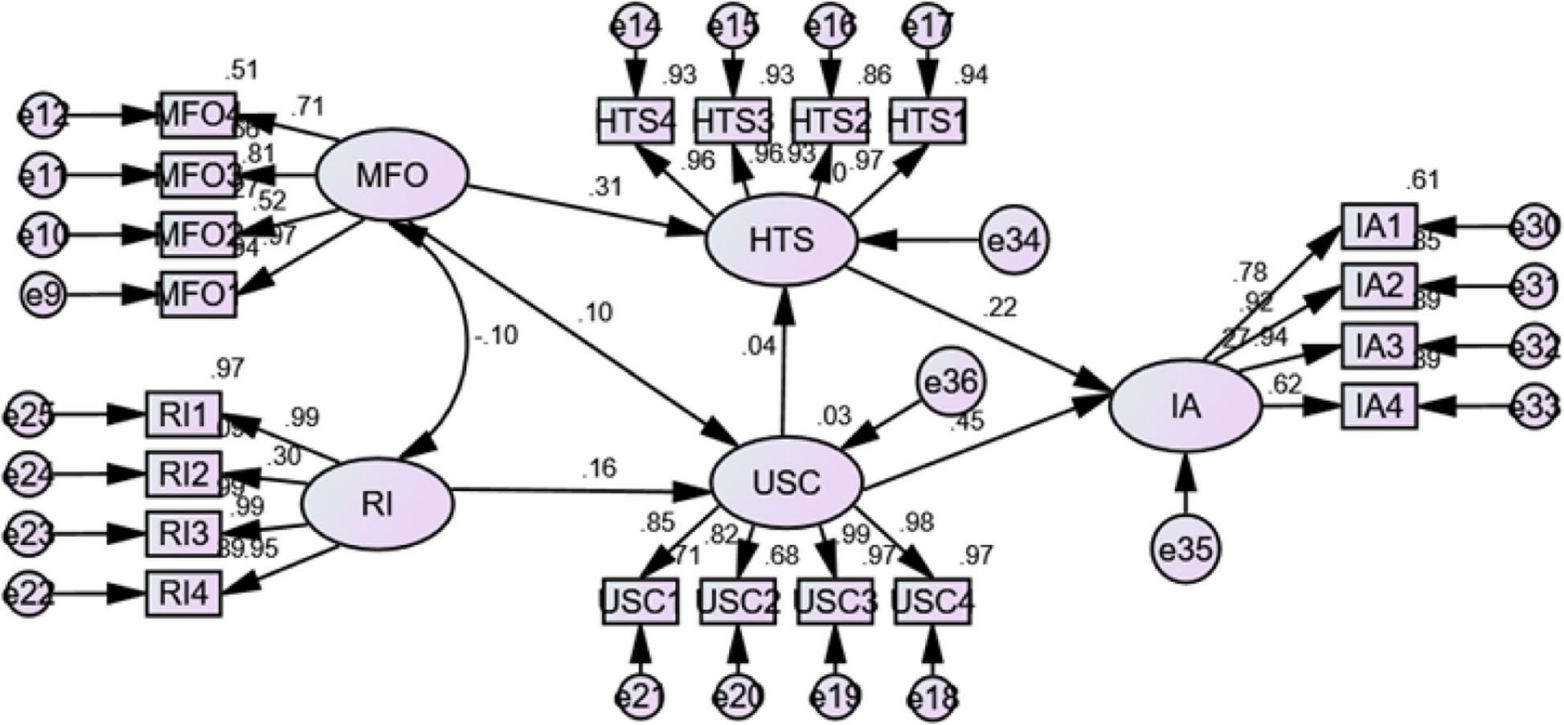
Healthcare Workers’ Structural Equation Model. (Legend: MFO - Multi-sectorial engagement. HTS - Adequate human resources, training and technical support. RI - Available and reliable infrastructure. USC - Usefulness, security and socio-cultural concerns. IA - Intention to Adopt. “e’s” - (i.e., e1, e2, e3, etc.) are the error terms of the variables

Based on the model fit indices (Table 2), the structural equation modelling found that all question items were acceptable Fig. 2.

**Table 2:** Results of the model fit indices

Test	Result	Acceptance criterion
Chi-square to the degrees of freedom CMIN/DF	1.333	< 2 or 3 [38, 45]
Root Mean Square Residual (RMR)	0.029	< 0.08 [46, 47]
Goodness of fit index	0.840	> 0.80 [47]
Normed fit index	0.912	> 0.90 [48]
Incremental fit index	0.977	> 0.90 [47]
Comparative fit index	0.976	> 0.93 [48]
Tucker Lewis index	0.972	> 0.95 [45, 47]
Root mean square error average	0.057	< 0.06 [49]

#### Development of a modified model

**Fig. 3:**
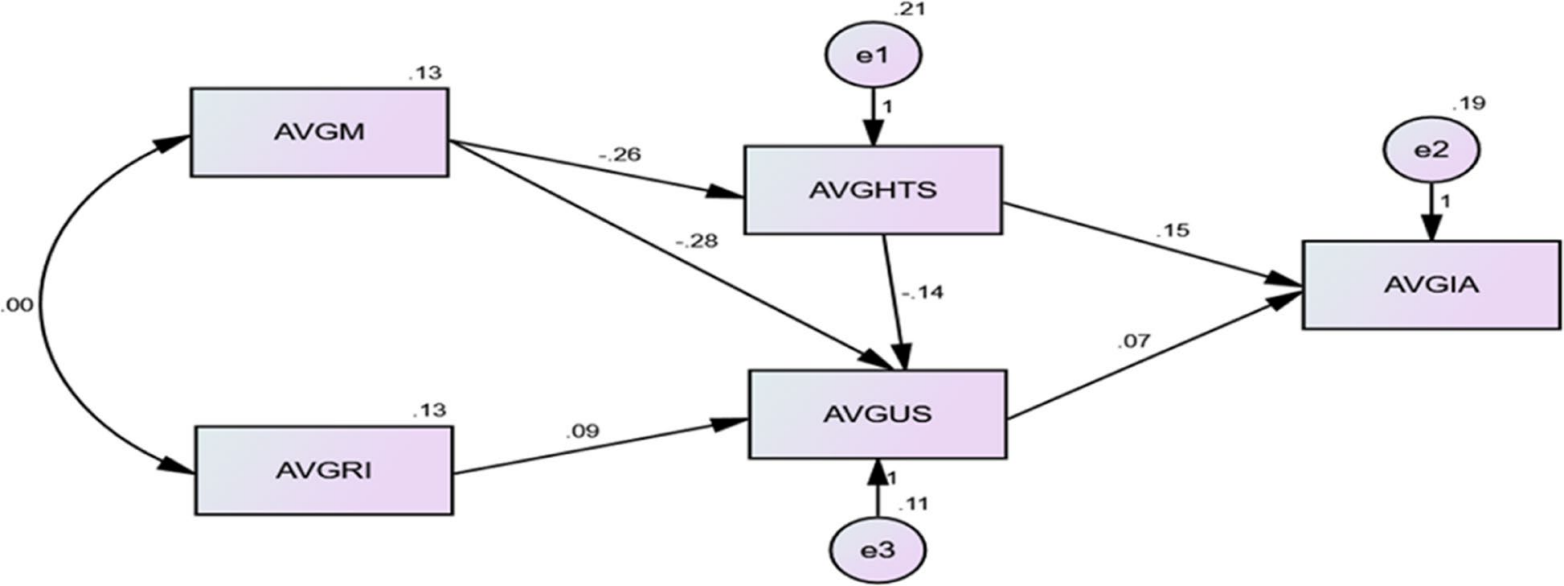
Modified Unified Healthcare Workers’ Model. AVGM - Multi-sectorial engagement, funding and ownership; AVGHTS - Adequate human resource, training and technical support; AVGRI - Available and reliable infrastructure; AVGUS - Usefulness, security and socio- cultural concerns; AVGIA - Intention to Adopt

Hoyle and Isherwood [45] and Kline [46] suggested that a publication based on SEM should include a modified model. Therefore based on the fit indices from the Structural Equation Model (HmAIM) (Table 2), a modified model was then developed using the averages of individual construct items as shown in Fig. 3. The model fitting indices show that the ratio of relative chi-square (CMIN) to the degrees of freedom (DF) was 1.152, considered acceptable since it was less than 2 [46, 47]. The RMR value for the model was 0.06 also considered acceptable as it was less than 0.08 [49, 50]. The goodness of fit index (GFI) for the model was 0.991, considered acceptable when above 0.80 [50]. The comparative fit index (CFI) value was 0.899 which although not> 0.9, met the requirements of Baumgartner and Homburg [51] and Doll et al. [52] being > 0.80. The Tucker Lewis index (TLI) was 0.883, close to 0.90, and was considered acceptable [53]. The Incremental Fit Index (IFI) was 0.905 and as it exceeds 0.90 was acceptable [49]. The Root Mean Square Error Average (RMSEA) for the modified model was 0.076 and considered acceptable being less than 0.08 [54].

All of the indices for the model were within acceptable limits and almost all the covariances were within the + 2.00 and − 2.00 assumption rule [55]. These findings demonstrate the model is fit for examining the causal effect between the constructs and can be applied to a much larger sample or the general population.

**Fig. 4:**
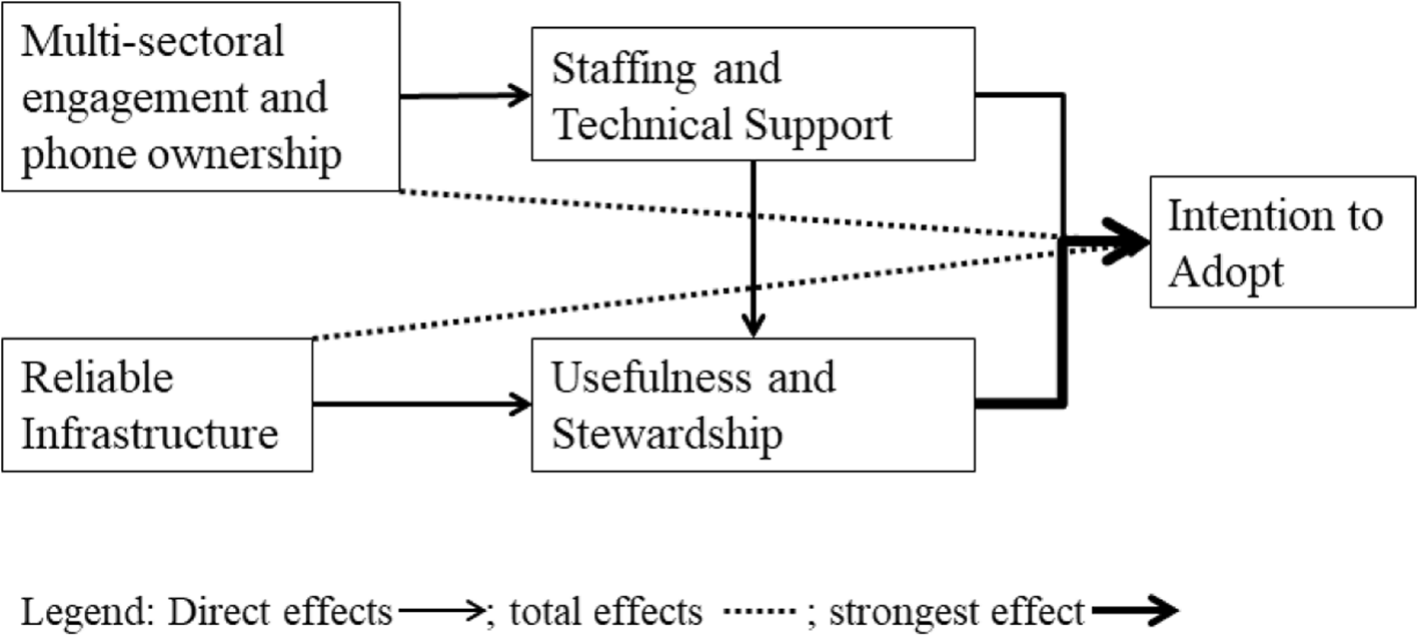
Healthcare Worker mHealth Adoption Impact Model (HmAIM)

The final model for the healthcare workers’ adoption of mHealth in the developing world is therefore presented in Fig. 4.

#### Findings from the HmAIM

Given that the chi-square to degrees of freedom value of the modified model is smaller than that of the actual model (i,e. 1.152 < 1.333), it shows that the hypothesised model (HmAIM) is able to adequately reproduce the observed sample statistic and is consistent with the characteristics of the observed sample [54]. The results show that there is no correlation between ‘multi-sectoral engagement and phone ownership’ and ‘reliable infrastructure’. There were direct and total effects realised from the HmAIM (Fig. 4). Direct effects were ‘staffing and technical support’ on ‘intention to adopt’; ‘usefulness and stewardship’ on ‘intention to adopt’; ‘multi-sectorial engagement and phone ownership’ on ‘staffing and technical support’; and ‘reliable infrastructure’ on ‘usefulness and stewardship’. There were also total effects such as ‘multi-sectoral engagement and phone ownership’ on ‘intention to adopt’; and ‘reliable infrastructure’ on ‘intention to adopt’. The standardised regression weight from ‘usefulness and stewardship’ to ‘intention to adopt’ is the greatest. The analysis therefore shows that there is a positive correlation among the latent variables in the model, with the strongest existing between ‘usefulness and stewardship’ and ‘intention to adopt’.

## Discussion

The paper describes the design and development of a valid and reliable instrument (HmAQ) and associated model (HmAIM) to identify factors that impact healthcare workers’ mHealth adoption in the developing world. HmAQ, is an evidence-based [36] and validated tool that was shown to be highly reliable, as determined by internal reliability (Cronbach alpha value of 0.82), and with good individual item correlation leading to retention of all items. The HmAQ has been shown to have strong construct validity, and the internal consistency of the five sub scales is high.

The results from the principal component analysis identified that the most important components influencing the adoption of mHealth by healthcare workers were availability of appropriate training and technical support, motivation, and adequate staffing. Collaboration and funding was the least important component influencing healthcare workers adoption of mHealth. Pilot testing of HmAQ using nurses and midwives, in the field and currently using an MoTeCH mHealth technology, confirmed its utility. All the items showed a good correlation, and all were retained.

The HmAQ differs from TAM and UTAUT, two of the most widely used models for technology assessment. Although applied within healthcare, TAM and UTAUT were not primarily developed for the assessment of healthcare systems but for explaining what factors were associated with email and word processing [11, 56], or database and accounting system use, [14] respectively. TAM and UTAUT also have noted limitations. For example, Ward [57] found that issues that affect health worker’s information technology / information system acceptance do not only revolve around the individual’s personal decision to use the technology, which is the main focus of TAM and UTAUT, but also on the organisational setting within which the system is deployed, in addition to the sociocultural and emotion factors. Similarly TAM and UTAUT have been criticised for their focus on settings where use of technology is voluntary unlike many healthcare settings [34], which this study addresses by using empirical findings from actual mHealth implementations. Based on the findings, we conclude that policy makers and managers of health institutions need to address issues of multi-sectoral engagement and phone ownership, improved staffing and technical support, reliable infrastructure, and enabling system’s utility to increase healthcare workers mHealth adoption in the developing world. This study has provided a viable alternate model which reliably assesses adoption of mHealth by healthcare workers in the developing world and could be used to provide evidence for the improvement and scaling up of mHealth initiatives.

### Limitations

Although this research provides an understanding of factors that affect mHealth adoption from the healthcare workers’ perspective, further research will be required to understand new dimensions of mHealth adoption as a result of emerging technology needs and new complexities in the healthcare work setting. Future research could look at increasing the number of participants, changing a sampling strategy (such as investigating mHealth interventions from the perspective of other stakeholders and potential users), or changing the research setting to observe the relationship among constructs. Further research could also be carried out to investigate how different cadres of healthcare workers respond to the questionnaire.

### Contributions

The study extends the body of knowledge by providing an alternate theoretical basis for understanding why health workers would either adopt or not adopt mHealth in the developing world. It identifies alternate factors for explaining and understanding adoption and use of mHealth. This will encourage future development of improved mHealth adoption models that consider broader and empirically identified issues affecting mHealth adoption. Thus the study findings will be of value to policy-makers and implementers alike, and contribute to more successful adoption and implementation of mHealth solutions reducing the occurrence of ‘pilotitis’. As suggested by Shachak et al. [58], to address the complexity of adoption issues in the health systems setting, it will be necessary to shift attention to a “multi-dimensional approach.” This research represents one example of a ‘multi-dimensional approach’.

## Conclusions

A reliable and valid tool (HmAQ) consisting of five constructs and 20 items, and its associated model (HmAIM) has been developed for investigating factors that influence adoption of mHealth by healthcare workers in the developing world. The HmAQ identified that the availability of appropriate training and technical support, motivation, and adequate staffing are the most important factors influencing the adoption of mHealth by healthcare workers. HmAIM presents a model for healthcare workers’ mHealth adoption with strong relationships among the constructs. Use of HmAQ would help guide policy-makers, researchers, and implementers as they implement mHealth solutions for healthcare workers in the developing world.

## Supplementary Information

**Additional file 1.**

## Data Availability

The datasets generated and/or analysed during the current study are not publicly available due to privacy concerns but are available from the corresponding author on reasonable request.
