# Supplementary material for: A healthcare workers’ mHealth adoption instrument for the developing world
Source: BMC Health Serv Res. 2022 Oct 2;22:1225. doi: 10.1186/s12913-022-08592-0 (PMC9526526; doi:10.1186/s12913-022-08592-0)
Supplement: Supplementary file 1 — Additional file 1. [file 12913_2022_8592_MOESM1_ESM.docx]

APPENDIX 1

|  | | Component | | | | | | |  |  |
| --- | --- | --- | --- | --- | --- | --- | --- | --- | --- | --- |
|  |  | 1 | 2 | | 3 | 4 | | 5 | Mean | SD |
| HTS4: Receiving requisite training on mHealth system use promotes adoption. | | .954 |  | |  |  | |  | 5.38 | .527 |
| HTS1: Availability of adequate health personal to provide mHealth services promotes adoption. | | .952 |  | |  |  | |  | 5.38 | 0.545 |
| HTS3: Reliable system support and phone maintenance promotes adoption. | | .945 |  | |  |  | |  | 5.38 | 0.527 |
| HTS2: Increase in my existing workload due to mHealth use affects adoption. | | .922 |  | |  |  | |  | 5.38 | 0.508 |
| USC4: Designing mHealth to reflect a health worker’s normal working environment promotes adoption. | |  | .926 | |  |  | |  | 6.32 | 0.562 |
| USC3: Designing mHealth to reflect the sociocultural setting for a particular group of people promotes adoption. | |  | .924 | |  |  | |  | 6.30 | 0.573 |
| USC1: Flexibility of the mHealth user-interface affects adoption. | |  | .898 | |  |  | |  | 6.34 | 0.601 |
| USC2: Protection against the unauthorized access of mHealth data increases adoption | |  | .861 | |  |  | |  | 6.27 | 0.578 |
| IA3: My intention to adopt mHealth will be as a result of the availability of reliable infrastructure | |  |  | | .865 |  | |  | 5.70 | 0.749 |
| IA2: My intention to adopt mHealth will be as a result of the availability of adequate human resources, training and technical support | |  |  | | .864 |  | |  | 5.75 | 0.760 |
| IA1: My intention to adopt mHealth will be as a result of the availability of multi-sectorial engagement and funding | |  |  | | .826 |  | |  | 5.81 | 0.751 |
| IA4: My intention to adopt mHealth will be as a result of the availability of user friendly systems, data security, and systems reflecting sociocultural concerns. | |  |  | | .769 |  | |  | 5.88 | 0.832 |
| RI1: Accessibility of mHealth services will affect adoption. | |  |  | |  | .970 | |  | 5.94 | 0.822 |
| RI3: Sustainability of electric power supply will affect adoption. | |  |  | |  | .964 | |  | 5.92 | 0.844 |
| RI4: Availability of mobile devices to use for mHealth affects adoption. | |  |  | |  | .954 | |  | 5.91 | 0.849 |
| RI2: Reliability of network facility supporting service will affect adoption. | |  |  | |  | .463 | |  | 5.47 | 0.750 |
| MFO1: Effective coordination between national, regional, and district health systems in delivering the mHealth will promote adoption | |  |  | |  |  | | .920 | 5.83 | 0.853 |
| MFO3: Ownership of mHealth by community and local stakeholders promotes adoption | |  |  | |  |  | | .844 | 5.85 | 0.694 |
| MFO4: A subsidized or totally waved cost on the mobile device for mHealth will affect health workers’ adoption. | |  |  | |  |  | | .792 | 6.00 | 0.836 |
| MFO2: Ability of patients to own personal mobile devices for mHealth services promotes health workers’ adoption. | |  |  | |  |  | | .654 | 5.71 | 0.692 |
| EigenValues. | | 5.38 | 4.04 | | 3.00 | 2.45 | | 1.34 |  |  |
| Variance Explained (%) | | 26.9 | 20.2 | | 15.0 | 12.2 | | 6.7 |  |  |
| Cronbach Alpha (%) | | 95.0 | 92.0 | | 88.8 | 88.0 | | 84.2 |  |  |
| Total Variance Explained (%) | | 81.1 |  | |  |  | |  |  |  |
| Total Reliability of Instrument (%) | | 83.6 |  | |  |  | |  |  |  |
| Kaiser-Meyer-Olkin Measure of Sampling Adequacy. | | | | 0.777 | | |  |  |  |  |
| ’Bartlett’s Test of Sphericity | Approx. Chi-Square | | | 2253.951 | | |  |  |  |  |
|  | df | | | 190 | | |  |  |  |  |
|  | Sig. | | | .000 | | |  |  |  |  |

**Appendix 2**

Convergent Validity

| *CONSTRUCTS* | *AVE* | *CR* | *Convergent Validity* |
| --- | --- | --- | --- |
| HTS | 0.913 | 0.937 | Established |
| IA | 0.684 | 0.894 | Established |
| MFO | 0.563 | 0.829 | Established |
| USC | 0.833 | 0.912 | Established |
| RI | 0.733 | 0.907 | Established |

Discriminant Validity

| **Correlation between constructs** | **Factor Loading (r)** | **Square Factor Loading (r^2^ sqr)** | **AVE1 AVE2**  **(AVEs SHOULD > r^2^)** | **Discriminant Validity** |
| --- | --- | --- | --- | --- |
| MFO <--> HTS | 0.319 | 0.1017 | 0.563 0.913 | Established |
| HTS <--> IA | 0.250 | 0.0625 | 0.913 0.684 | Established |
| HTS <--> USC | 0.063 | 0.0039 | 0.913 0.833 | Established |
| HTS <--> RI | 0.069 | 0.0047 | 0.913 0.684 | Established |
| MFO <--> USC | 0.088 | 0.0014 | 0.563 0.833 | Established |
| MFO <--> IA | 0.037 | 0.0013 | 0.563 0.684 | Established |
| MFO <--> RI | 0.102 | 0.0104 | 0.563 0.733 | Established |
| RI <--> IA | 0.061 | 0.0037 | 0.733 0.684 | Established |
| USC <--> RI | 0.152 | 0.0231 | 0.833 0.733 | Established |
| USC <--> IA | 0.468 | 0.2190 | 0.833 0.684 | Established |

Appendix 3. Structural Equation Model indices

| **Model** | **NPAR** | **CMIN** | **DF** | **P** | **CMIN/DF** |
| --- | --- | --- | --- | --- | --- |
| Default model | 50 | 209.803 | 160 | .005 | 1.331 |
| Saturated model | 210 | .000 | 0 |  |  |
| Independence model | 20 | 665.118 | 190 | .000 | 3.501 |
|  |  |  |  |  |  |
|  |  |  |  |  |  |

| Model | NFI | RFI | IFI | TLI | CFI | RMR | GFI | RMSEA |
| --- | --- | --- | --- | --- | --- | --- | --- | --- |
| Default model | .912 | .896 | .977 | .972 | .976 | 0.029 | 0.840 | 0.057 |
